# Supplementary material for: Differential Evolution approach to detect recent admixture
Source: BMC Genomics. 2015 Jun 18;16(Suppl 8):S9. doi: 10.1186/1471-2164-16-S8-S9 (PMC4480842; doi:10.1186/1471-2164-16-S8-S9)
Supplement: Additional file 1 — Supplementary materials. Selection of the optimal number of components. [file 1471-2164-16-S8-S9-S1.pdf]

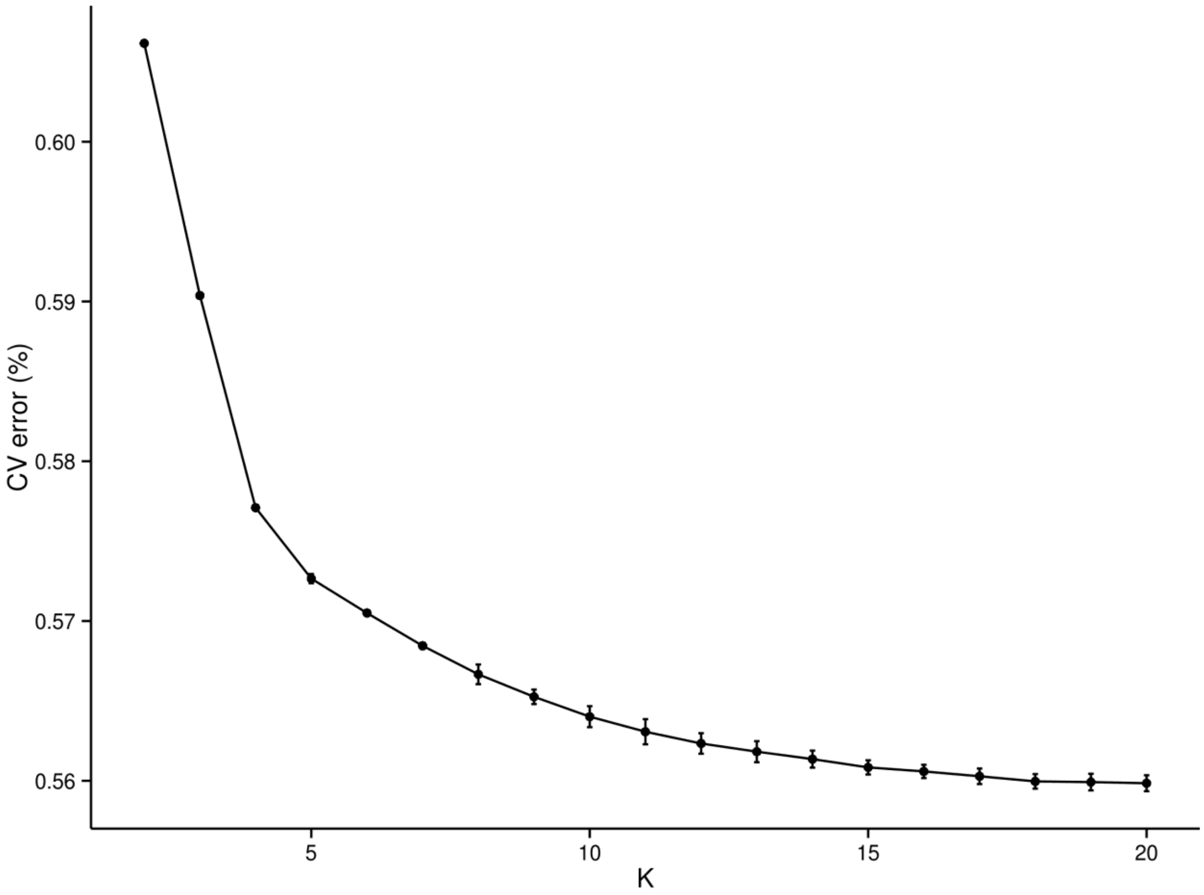

Supplementary Figure 1: Selection of the optimal number of admixture components, ten-fold cross-validation (CV), K=2..20.

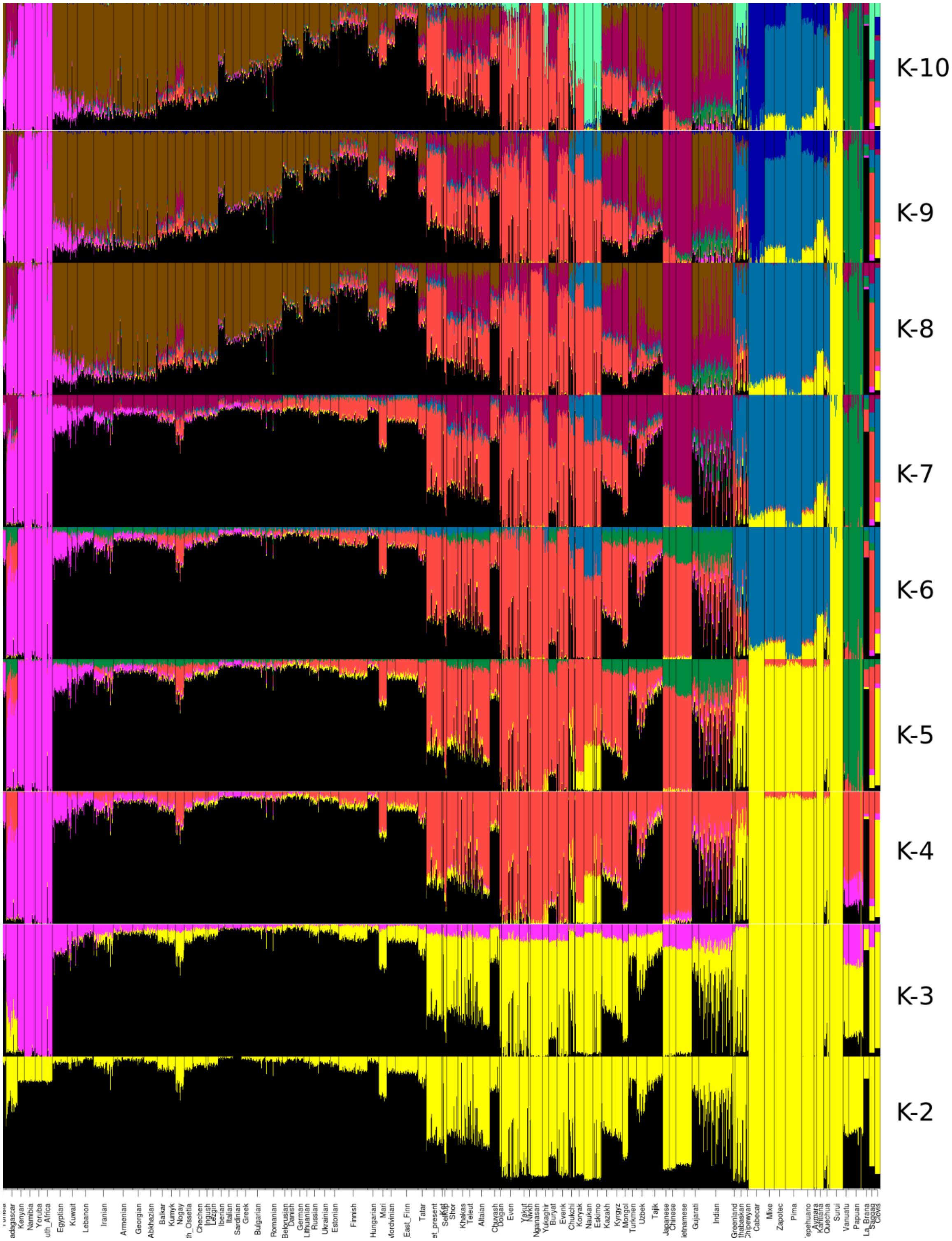

Supplementary Figure 2: Selection of the optimal number of admixture components, K=2..10.

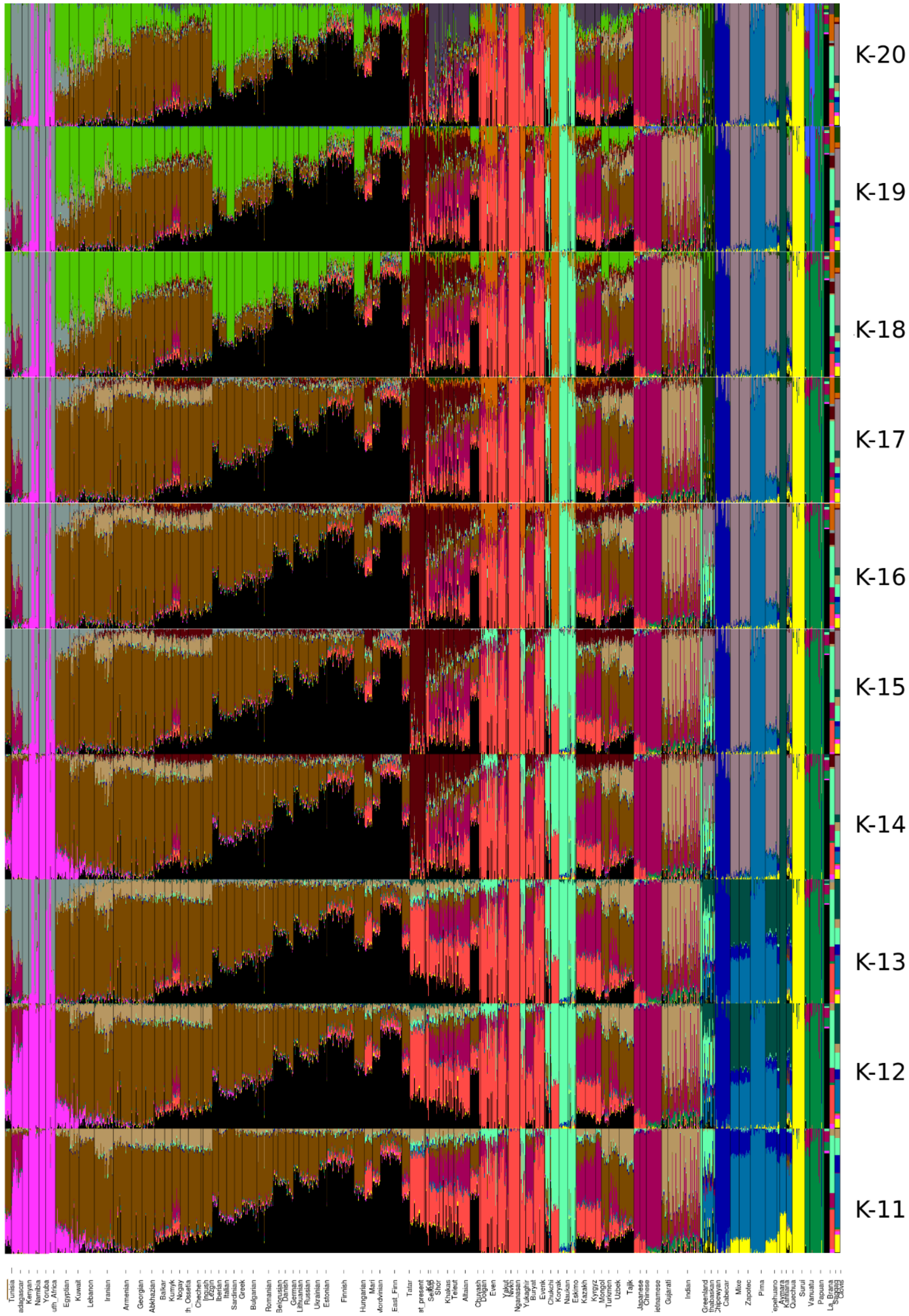

Supplementary Figure 3: Selection of the optimal number of admixture components, K=11..20.
